# Fabrication of N-doping activated carbons from fish waste and sawdust for Acid Yellow 36 dye removal from an aquatic environment

**DOI:** 10.1038/s41598-023-33075-5

**Published:** 2023-04-11

**Authors:** Mohamed A. El-Nemr, Mohamed A. Hassaan, Ibrahim Ashour

**Affiliations:** 1 Department of Chemical Engineering, Faculty of Engineering, Minia University, Minia, 61519 Egypt; 2 Environment Division, National Institute of Oceanography and Fisheries (NIOF), Kayet Bey, El-Anfoushy, Alexandria, Egypt

**Keywords:** Environmental chemistry, Chemistry

## Abstract

Acid Yellow 36 (AY36) dye is a synthetic azo dye that is excessively used in various industries, causing hazardous environmental effects. The main target of this study is the preparation of self-*N*-doped porous activated carbon (NDAC) and the investigation in eliminating the AY36 dye from the water solution. The NDAC was prepared by mixing fish waste (60% protein content) which was considered a self-nitrogen dopant. A combination of Fish waste, sawdust, zinc chloride and urea with a mass ratio (5:5:5:1) was submitted to hydrothermal process at 180 °C for 5 h followed by pyrolysis for 1 h under N_2_ stream at 600, 700, and 800 °C. Fabricated NDAC was qualified as an adsorbent for recovering AY36 dye from water using batch trials. The fabricated NDAC samples were characterized by FTIR, TGA, DTA, BET, BJH, MP, *t*-plot, SEM, EDX, and XRD methods. The results showed the successful formation of NDAC with nitrogen mass percentage content (4.21, 8.13 and 9.85%). The NDAC prepared at 800 °C had the largest nitrogen content (9.85%) and was labeled as NDAC800. This later had 727.34 m^2^/g, 167.11 cm^3^/g, and 1.97 nm for specific surface area, the monolayer volume and the mean pores diameter respectively. By being the more efficient adsorbent, NDAC800 was chosen to test AY36 dye removal. Therefore, it is selected to investigate the removal of AY36 dye from aqueous solution by varying important parameters such as solution pH, initial dye concentration, adsorbent dosage and contact time. The removal of AY36 dye by NDAC800 was pH-dependent, with the optimum pH value 1.5 giving 85.86% removal efficiency and 232.56 mg/g maximum adsorption capacity (*Q*_m_). The kinetic data exhibited the best fit model with the pseudo-second-order (PSOM), while the equilibrium data fit well with the Langmuir (LIM) and Temkin (TIM). The mechanism of AY36 dye adsorption may be ascribed to the electrostatic contact between the dye and the available charged sites on NDAC800 surface. The prepared NDAC800 may be considered as an efficient, available, and eco-friendly adsorbent for AY36 dye adsorption from simulated water.

## Introduction

Nowadays, the insufficiency of clean and fresh water resources becomes the biggest obstacle in order to meeting the progress of the civilization of humanity. Persistent pollution is considered a major factor that threatens the world's water resources. Many attempts have been made to create solutions to compensate the shortage of various water resources, but these attempts are still quite limited. So, it became necessary to link scientific research with all types of water treatment.

One of the most toxic pollutants is organic dyes which are harmful to living things and the environment. Acid Yellow 36 dye is a mono azo dye, water-soluble, pH indicator and has various applications such as leather, paper and textile industries^1–4^. Many treatment procedures are utilized for water treatment, depending on the type of treated objectives^5^. Generally, methods for water treatment processes are adsorption^6^, oxidation procedures (photocatalysis)^7–9^, precipitation^10^, coagulation/flocculation^11^, reverse osmosis^12^, ion exchange^13^, filtration^13^, biological treatment^14^ and advanced oxidation processes^15–19^. Adsorption technique is the most effective water purification process due to its simplicity, effectiveness and eco-friendly advantage of the absence of generated sludge^20–24^. Adsorption method owned many advantages such, as its low cost, appropriateness and effortlessness of process and designs^25,26^, ease of use, low energy requirements, and lack of production of harmful side effects from conventional treatments^11^. Due to their accessibility, ease of preparation, and exceptional adsorption capacity findings, activated biomass-based treatments are thought to have received recently more attention among the numerous available adsorbents for dyes removal from liquid effluents^25–27^.

Especially, various technological systems for removing dyes were examined such as the photocatalytic degradation using ZnO as a photocatalyst^28^, reverse micelles^29^, Photoelectro-Fenton using iron electrodes^30^, adsorption onto green nano-ceria, and NH_2_ functionalized green nanoceria^31^, Orange peels, and Rice Husk^32^, and Pinecone^33^, solvent extraction using the carrier trioctylamine^34^ and electrocoagulation using iron electrodes^35^.

One of the best methods for improving activated carbon's adsorption effectiveness is nitrogen doping, which increases its electronic conductivity, adds additional ion-storage sites, and reduces amplitude because the nitrogen atom has one more electron than the carbon atom^20–23,36^. The doped nitrogen atoms within the six-membered carbon ring create local stress when it is added to a carbon material, which causes the deformation of the original carbon structure^37^.

The lone pair of electrons that nitrogen atoms carry would delocalize the initial sp^2^ hybrid electron cloud on the carbon skeleton, improving electron transportation and reactivity of the activated carbon surface since nitrogen atoms have a higher electronegativity (3.04) than carbon atoms (2.55). Simultaneously, doped nitrogen atoms can significantly enhance carbon compound adsorption by giving additional free electrons^23^. Recent studies showed that doping-activated carbons with a specified amount of nitrogen, sulphur, or phosphorus enhance their surface stability as well as a portion of their pseudo capacitance, boosting their overall specific capacity^38–40^. Surface groups on activated carbons have a significant role in defining the electrochemical interface state as well as the properties of carbon surfaces. These qualities include stability, isoelectric point, contact resistance, ion adsorption, and self-discharge, among others^41^. The existence of efficient heteroatom groups on the surface of activated carbons is expected to enhance their ion adsorption capacity as well as their hydrophilicity/lipophilicity. As a result, it is believed that modifying the surface properties of carbon materials is good policy to boost efficiently the adsorption capacity of carbon-based materials. Nitrogen doping in carbon matrixes can take several forms. There are two types of doping: post-treatment doping (PTD) and in-situ doping (ISD) as reported in the literature^37^. Chemical vapor deposition (CVD), solvent-thermal synthesis, and laser ablation are examples of ISD processes. PTD procedures include plasma therapy, irradiation, and thermal annealing at high temperatures. A more advanced and less-doped method than post-treatment doping is the ISD of nitrogen-rich precursors. Therefore, there has been a lot of interest in producing of high nitrogen-doped activated carbons (NDACs) following PTD way^42^. NDACs have been widely used in numerous fields to improve their characteristics and mobility by nitrogen-doping activated carbon. Recently, nitrogen-doped carbon compounds have been identified as potential materials for heterogeneous catalysis, such as catalytic hydro-de oxygenation, which is a key step in refining bio-oil before creating high-value chemicals or transportation fuels^43^. Nitrogen doping protects the pores structure of carbon materials and improves their electrochemical characteristics, making them suitable for use as supercapacitors^44–47^. Additionally, the metal contained within a nitrogen-doped carbon nanosphere was used as a photocatalyst, opening new avenues for environmental pollution remediation. In general, photocatalysis using solar energy is viable for addressing energy shortages and pollution^48^. With a large specific surface area, NDACs could be excellent materials for removing contaminants such as toxic pollutants from wastewater^24^. Furthermore, NDACs can be used as an electro-catalyst in manufacturing^49^. Similarly, activated carbon doped with nitrogen can be employed as a novel material with a high CO_2_ collection capacity^50^.

El-Nemr et al.^21^ developed modified biochars from pea Pisum sativum using NH_2_ and TETA and assessed its use for enhancing the adsorption capacity of Acid Orange 7 (AO7) dye. The removal of AO7 dye using the modified (PeaNH_2_ and Pea- TETA) biochars derived from the pea Pisum sativum peels were investigated. The adsorption capacity at equilibrium (q_e_) of the modified biochar was significantly enhanced from 78.18 for unmodiefied biochar to 523.12 mg/g for Pea- TETA biochar at initial concentration 300 mg/L and dosage 0.50 g/L. The percentage removal of AO7 dye reached 99% for Pea- NH_2_ and 98% for Pea-TETA. Sun et al.^49^ fabricated nitrogen self-doped porous activation carbon (N-PAC) with large specific surface area (SBET = 1300.58 m^2^/g) as bifunctional electrocatalysts. The N-PAC is prepared by a simplistic pyrolysis process from marine algae at controlled temperature with ZnCl_2_.

Thirunavukkarasu et al.^31^ use the green nanoceria synthesized from *Prosopis juliflora* (Sw.) leaves extract, which amine functionalized to GN-NH_2_ (AGN) with epichlorohydrin and ammonium hydroxide. In the ambient temperature and at acidic pH of 2, the maximum removal efficiency of AY36 is 92.9% for AGN respectively. The kinetic data exhibited good correlation coefficient (r^2^ > 0.99) for the pseudo-second-order and elovich models with marginal error values signifying the chemisorption type of adsorption process. The maximum adsorption capacities of AGN was 26.95 (mg/g).

The main goal of this paper is to focus on and describe a novel technique for the fabrication of nitrogen self-doping activated carbon (NDAC) with a high nitrogen content at different temperature with ZnCl_2_ as (activation reagent) and investigate its effects on the removal of AY36 dye from water.

## Materials and methods

### Chemicals and equipments

Ethanol (C_2_H_5_OH), urea (NH_2_CONH_2_), Zinc chloride (ZnCl_2_), and Acid Yellow 36 dye (AY36) (Molecular formula: C_18_H_16_N_3_NaO_3_S; Molar Mass: 377.39 g/mol; *λ*_max_: 450 nm) were obtained from Sigma Aldrich, USA. Sawdust was obtained from a local carpenter, Alexandria, Egypt. Fish waste (mixture of *Atherina hepseetus* and *Sardina Pilchardus* Fishes waste of 60% protein) was obtained from the NIOF, Egypt. HCl, (30–34%) was obtained from (SD-FCL), Mumbai, India. UV–visible spectrophotometer (Analytic Jena, model SPEKOL1300) has glass cells of 10 mm optical path used for dye concentration determination. Shaker (A JS shaker, model JSOS-500), Thermo shaker incubator (GSSI-100T sh), Nabertherm B180 Tubular Furnace (RT 50/250/13), and pH meter JENCO (6173) were used for the experimental work. Fourier transform infrared spectrometer (FT-IR: Bruker Vertex 70 connected to Platinum ATR model V-100). X-ray Photoelectron Spectroscopy (XPS) was performed using a Thermo Fisher Scientific K-Alpha XPS with a pass energy of 50 eV at a base pressure of ~ 10–9 mbar.

The BET surface area (*S*_BET_) studies of the NDACs were done using N_2_ adsorption–desorption analysis at 77 K using an analyzer instrument (BELSORP—Mini II, BEL Japan, Inc.) The BET^51–53^, analysis of the adsorption–desorption isotherm curve was used to calculate the monolayer volume (*V*_m_) (cm^3^ (STP)/g), the specific surface area (*S*_BET_) (m^2^/g), the volume of the total pore (*V*_T_) (p/p_0_) (cm^3^/g), mean pore diameter (nm) and energy constant (C). The following equation was used to determine the average pore-radius (1):

1 $$r\left( {nm} \right) = \frac{{2V_{T} \left( {mLg^{{{-}1}} } \right)}}{{a_{s,BET} \left( {m^{2} g^{{{-}1}} } \right)}} \times 1000$$

Using the Barrett-Joyner-Halenda (BJH) methodology, the BELSORP analysis programme software was also used to calculate the micropore surface area (*S*_mi_) and micropore volume (*V*_mi_), as well as the mesopore surface area (*S*_mes_) and mesopore volume (*V*_mes_), of biochar. The pore size distribution (PSD) was calculated using the BJH approach from the isotherm desorption curve^54^.

Thermal-gravimetric analyses (TGA) were performed using the SDT650 instrument in a temperature range of 25–1000 °C, at 10 °C per minute, as a temperature ramp under 100 mL/min flow of nitrogen gas^55^. Scanning Electron Microscope (SEM) examination was carried out utilizing a Quanta 250 FEG SEM instrument with 500 kV HV, and large-field low vacuum SED (LED) coupled with EDX unit to analyze the surface-morphology and porosity of the NDACs. D2 PHASER Instrument, manufactured by Bruker in Germany, was used for the XRD analysis.

### Preparation of self-Nitrogen doped activated carbon (NDAC)

**Figure 1:**
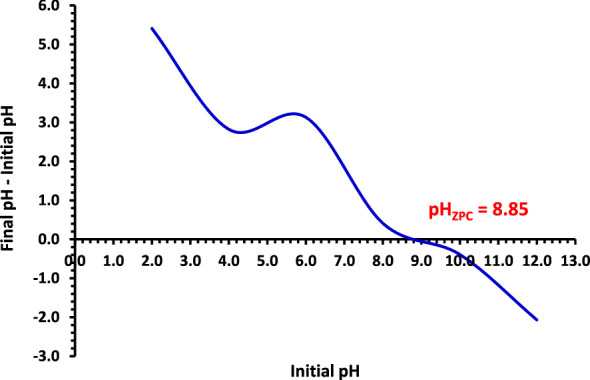
The pH_ZPC_ determination of the NDAC800 at 25 ± 2 °C.

The NDACs were prepared by a uniform mixing of sawdust with ZnCl_2_, Fish waste (60% protein) and urea in a mass ratio of 5:5:5:1 and distilled water (300 mL). The prepared mixture was transported to a 500 mL Teflon cup (1,1,2,2-polytetrafluoroethylene) and treated hydrothermally by placing it into a stainless steel autoclave at 180 °C for 5 h. This prepared fusion material was then dried at 125 °C for 24 h. The carbonization of the dried mixture to form NDACs was performed via pyrolysis in the high-temperature area of the tubular furnace under N_2_ stream rate of 100 mL/min. The pyrolysis process was realized at 600, 700 and 800 °C and kept constant at this temperature for 1 h. The obtained NDACs were refluxed in 2N HCl solution for 2 h, filtered, washed with EtOH followed by distilled water and oven dried overnight at 125 °C. Finally, the targeted NDACs were labeled as NDAC600, NDAC700, and NDAC800 (Fig. 1S).

### pH of point of zero charge (pH_PZC_)

The approach outlined in the literature was used to obtain the pH_PZC_^56,57^. In brief, in 100 mL flasks, 50 mg of NDAC800 was taken in 50 mL of 0.1 M NaNO_3_ solutions. The initial pH solution (pH_i_) was adjusted to a value ranged from 2 to 12 using 0.1 M HCl or NaOH and shaken 24 h. Then the final pH of the supernatant liquid (pH_f_) was calculated. Moreover, the variance at the beginning and final pHs (ΔpH = pH_i_ − pH_f_) was plotted against the pH_i_. The pH value at the ΔpH equalled zero was ascribed as pH_PZC_ of the adsorbent. The pHzpc value of NDAC800 was reported to be 8.85 (Fig. 1). The result designates that, below this pH value, the surface of the NDAC800 has a positive charge due to the protonation of nitrogen atoms into NDAC-H^+^_._

### Acid Yellow 36 dye adsorption

1.0 g/L stock solution of AY36 dye was prepared by dissolving 1.0 g of AY36 dye into 1 L distilled water. The removal investigation of AY36 dye was carried out using the batch equilibrium technique. In a shaker, 100 mL of AY36 dye solutions of various concentrations were was agitated with various amount (50 to 250 mg) of the produced NDAC. The solution was examined using a UV–visible spectrophotometer at λ = 450 nm for AY36 dye residual concentration determination. The adsorption capacities of nitrogen-doped activated carbon can be measured using Eq. (2):

2 $${q}_{t}=\frac{\left({C}_{0}-{C}_{t}\right)}{w}\times V$$

where *q*_t_ (mg/g), *C*_0_ (mg/L), and *C*_t_ (mg/L) are the capacity of adsorbent at time *t*, the initial concentration of dye; the residual concentration of the pollutant after adsorption had taken place over a period time *t*, respectively. *V* (L) and *W* (g) are the waste solution volume of dye in liters and the mass of NDAC in grams. The following equation is applied to measure the AY36 dye elimination % from the water solution (3):

3 $$Removal(\%)=\frac{\left({C}_{0}-{C}_{t}\right)}{{C}_{0}}\times 100$$

The impact of pH on AY36 dye removal was studied using 100 mg of NDAC800 and 100 mL of AY36 dye solutions (100 mg/L) by varying the initial pH values 1.5, 3, 5, 7, 9 and 11. The solution pH was adjusted using HCl (0.1 M) or NaOH (0.1 M) solutions. The mixtures were agitated at 200 rpm for 2 h at 25 ± 2 °C and the final AY36 dye was determined.

The impact of adsorbent dose and the isotherm study were performed using different concentrations of AY36 dye solutions (100–400 mg/L) using various weights of NDAC800 (50–250 mg) in 100 mL of AY36 dye solutions. Then the solutions were shaken at 200 rpm for 10, 15, 30, 45, 60, 90 and 120 min at 25 °C and the supernatants were analyzed spectrophotometrically.

## Results and discussion

### Morphology and structure description

#### FTIR analysis

**Figure 2:**
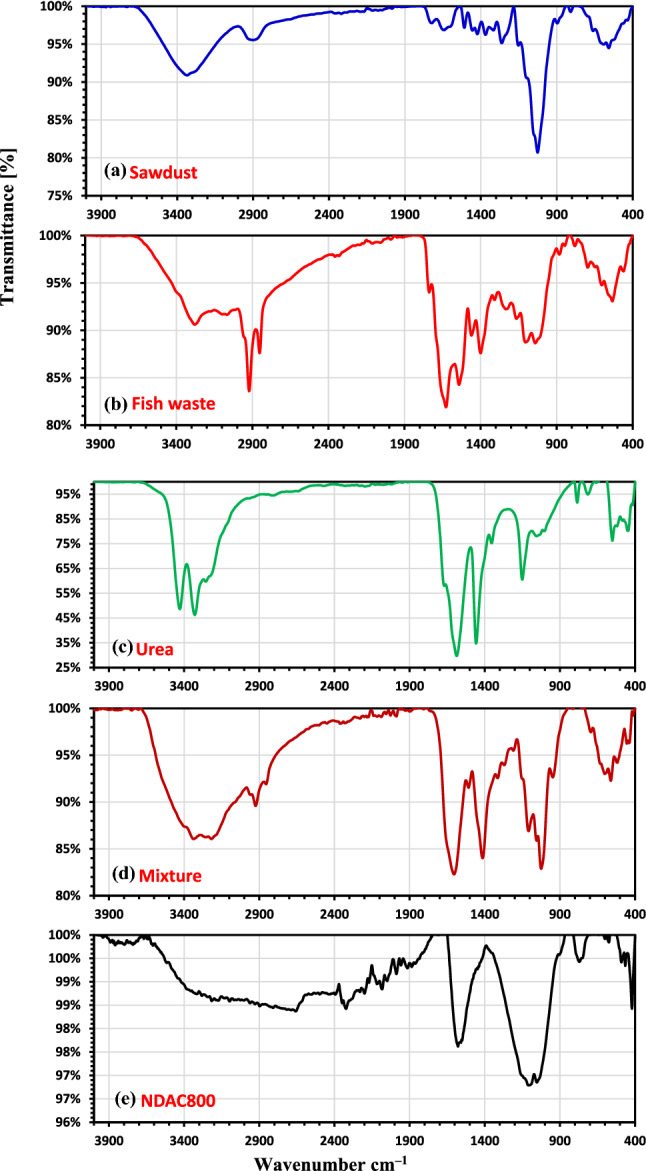
The FTIR studies of (**a**) raw sawdust; (**b**) raw fish waste materials of 60% protein; (**c**) urea; (**d**) the mixture (raw sawdust, fish waste, ZnCl_2_ and urea) hydrothermal; and (**e**) NDAC800.

The efficient groups of raw sawdust, fish waste, the mixture (sawdust, fish waste, ZnCl_2_ and urea) and NDAC800 were determined by FTIR and shown in Fig. 2a–e. The FTIR spectrum of sawdust (Fig. 2a) shows the appearance of an absorption broad peak at 3334.66/cm, proving the occurrence of free and intermolecular bonded –OH groups. The appearance of the peak at 2899/cm assigned to C–H stretching vibrations from –CH_2_ group, and the peak at 1723.59/cm corresponds to –C=O stretching vibrations from aromatic groups of lignin. The appearance of a peak at 1634.45/cm may be due to N–H amide group, and the peak at 1422.97/cm is due to –OH deformation. Another strong sharp peak at 1026.04/cm characterizes C–O stretching of the primary alcohol. At last, the peaks at 590.43 and 557.99/cm were related to the bending vibration modes of aromatic compounds (Fig. 2a). The FTIR spectrum of the fish waste (mixture of *Atherina hepseetus* and *Sardina Pilchardus*) is represented in Fig. 2b, which showed for amide A, two broad bands, one of them appeared at 3279.36/cm is due to OH while the other broad peak at 3066.73/cm is attributed to N–H stretching vibrations. The occurrence of two broad bands of amide B at 2924.18 and 2852.78/cm correspond to CH_2_ stretching vibrations. The peak of ester and lipids groups is observed at 1738.06/cm. Also, amide I, II, and III have appeared at 1627.3 (stretching vibration of C=O), 1541 (N–H bending and C–N stretching vibrations), 1308 and 1232.55/cm (N–H bending and C–N stretching vibrations and O=C–N), respectively. Finally, absorption peaks at about 1104.31, 1040.41, 603.61 and 533.73/cm assigned to asymmetric stretching of phosphate group (PO_4_^3−^). The FTIR of pure urea is shown in Fig. 2c. The N–H stretching peak appeared at 3428.85–3255.57/cm, and the C=O stretching peak appeared at 1669/cm. The N–H deformation stretching peak appeared at 1586.35/cm, while the C–N stretching peak appeared at 1457/cm. The FTIR of the mixture of raw sawdust, fish waste, ZnCl_2_ and urea was shown in Fig. 2d. The peak of O–H stretching for alcohol and the N–H stretching frequencies had become stronger, broader, and appeared at 3337.00 and 3218.61/cm, respectively. Occurrence of two broad bands at 2924.43 and 2855.47/cm are due to CH_2_ stretching vibrations of sawdust and amide B. The appearance of strong sharp wide peak at 1604.04/cm which representing the N–H amide group in sawdust, amid I in fish meal and in urea. The appearance of N–H bending and C–N stretching vibrations of amide II at 1507.46, while the C–N stretching peak in urea appears at 1414.21/cm. The FTIR analysis of NDAC800 was given in Fig. 2e. The broad set of peaks with low intensity appeared between 3745 and 3096.68/cm are corresponding to N–H stretching. The appearance of these peaks may be due to the hydrogenation of some of the nitrogen atoms. The C–H stretching has a broad low-intensity peaks appeared at 2897.11 and 2808.46/cm. The appearance of low-intensity peaks at 2394.36, 2348.46, and 2322.76/cm are assigned to C≡N stretching vibrations of isonitrile-cyano-terminal groups. The allene (C=C=C) and ketamine (C=C=N) groups appeared as a set of medium peaks at 2229.50–2048.54/cm. The band owing to C=C stretching vibration is performed at 1705/cm. The appearance of the strong sharp peaks, which relevant to sp^2^ structure of the carbon atoms with a higher shifting from 1508.57 to 1577.72/cm and from 1026.09 to 1107.12 and 1053.22/cm confirmed the successful formation of nitrogen doping activated carbon at 800 °C. This shifting may occur due to nitrogen doping which causes the disordered structure of the carbon network.

#### BET analysis

**Figure 3:**
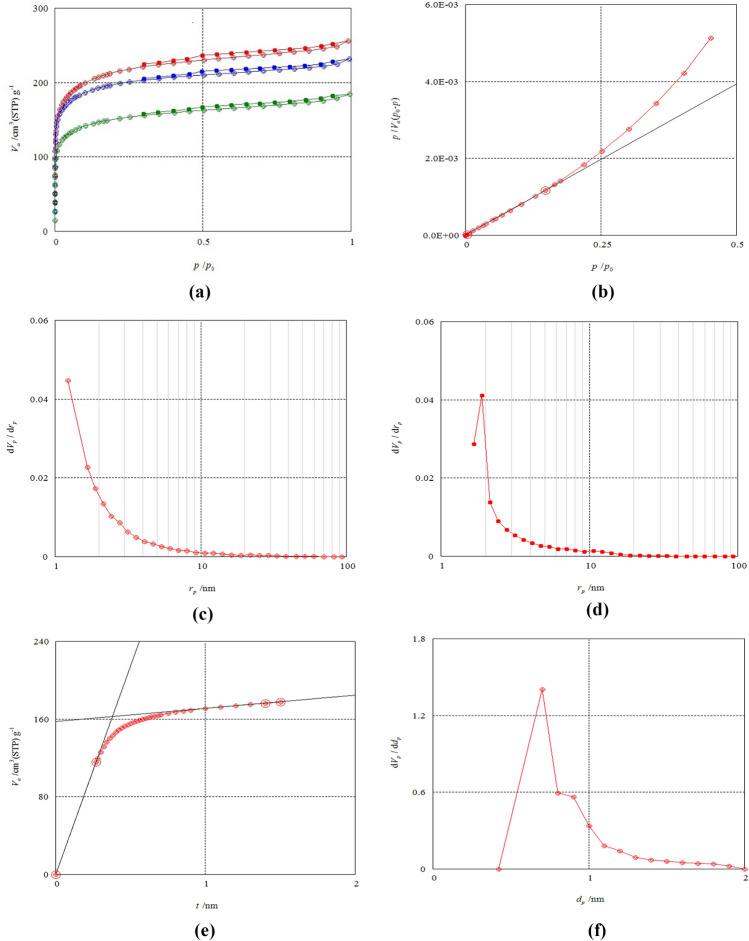
(**a**) Adsorption–desorption of NDAC600 (green), NDAC700 (red) and NDAC800 (blue); (**b**) BET analysis of NDAC800; (**c**) BJH analysis by adsorption of NDAC800; (**d**) BJH analysis by desorption of NDAC800; (**e**) *t*-plot analysis of NDAC800; (**f**) MP analysis of NDAC800.

The N_2_ adsorption–desorption isotherms of NDAC600, NDAC700 and NDAC800 are represented in Fig. 3a–f. These concluded that the adsorption isotherm of the various nitrogen NDACs are microporous typical type I and subsequently possessed a strong interaction between NDAC surface and adsorbate (Fig. 3a). According to BET analysis results (Table 1, Fig. 3b), NDAC600 has a surface area, monolayer volume, total pore volume, and mean pore diameter of 553.63 m^2^/g, 127.20, 0.2844 m^3^/g and 2.0584 nm, respectively while NDAC700 possessed the highest surface area of 783.78 m^2^/g, monolayer volume, mean pore diameter, and total pore volume of 180.08 cm^3^/g, 2.0194 nm, and 0.3957 cm^3^/g, respectively. The specific surface area of NDAC800 was 727.34 m^2^/g, the monolayer volume, total pore volume, and mean pore diameter were 167.11, 0.3576 cm^3^/g and 1.9668 nm, respectively. Observably, the mean pore diameter value of NDAC800 is marginally decreased than the other NDAC at 600 and 700 °C, indicating that the increase in temperatures from 600 to 800 °C may be slightly affected on the formation of micropore of NDAC800. The *t*-plot analysis reflected the correlation between the thickness of adsorption layer and relative pressure. The *t*-plot curves of NDAC600, NDAC700, and NDAC800 are shown in Fig. 3c. *t*-plot analysis of NDAC at 600, 700, and 800 °C showed type II, which meant that they possessed uniformly sized micropores. From Table 1, the pore surface area of NDAC prepared at 600, 700 and 800 °C are 640.649, 894.315 and 864.640 m^2^/g, respectively. The pore volume of NDAC at 600, 700 and 800 °C were 0.2441, 0.3515 and 0.3193 cm^3^/g, respectively, while the average pore diameter are 0.7573, 0.7813 and 0.7341 nm, respectively. The MP-conclusions plot's are quite similar to those of a t-plot because it is an analysis technique derived from one. The MP-plot is significantly impacted by the chemical differences between standard material and sample surface, as well as by the action of micropore filling, hence it cannot create a smooth curve. However, the MP-plot can be used to determine whether micropores exist and what size range they fall into. The MP-plot of NDAC at 600, 700 and 800 °C are shown in Fig. 3d, which shows that NDAC prepared at 600, 700, and 800 °C has micropores of 0.3–1.1 nm diameter, a maximum distribution peak at 0.5 nm while the distribution area of pores in NDAC800 is the largest one. Also, the surface area and the volume of pores were shown in Table 1. The pore surface area (a_1_–a_2_) of NDAC600, NDAC700 and NDAC800 was 604.981, 851.67 and 810.177 m^2^/g while the pore volume (*V*_P_) was 0.2536, 0.3626 and 0.3337 cm^3^/g, respectively. BJH analysis desorption and adsorption used to determine the distribution of the mesopores pore size. BJH analysis desorption–adsorption of NDAC at 600, 700 and 800 °C are shown in Fig. 3e, f and Table 1. From Fig. 3e, the NDAC600 has a 2.3–9.0 nm radius of mesopore, and it has a distribution peak at 3.0 nm. The NDAC700 has 2.5–10.0 nm radius of mesopores, and it has a distribution peak at 3.5 nm, while the NDAC800 has 2.2–9.0 nm radius of mesopores, and it has a distribution peak at 3.1 nm (Fig. 3e). The BJH desorption analysis of NDAC600, NDAC700, and NDAC800 had integrated pore volume (*V*p) 0.0512, 0.0609 and 0.0499 cm^3^/g, and the mesopore specific surface area 32.867, 40.589 and 32.378 m^2^/g, respectively (Table 1). The BJH adsorption analysis of NDAC600, NDAC700 and NDAC800 had integrated pore volume (*V*p) 0.0822, 0.1090 and 0.0862 cm^3^/g and the mesopore-specific surface area 76.743, 109.490 and 84.850 m^2^/g, respectively (Table 1). Generally, from the BJH desorption and adsorption results showed the mesopore specfic surface area represented a marginal amount of the total specfic surface of nitrogen doping activated carbons. The increase in temperature from 600 to 700 °C showed an increase in surface area, while a further increase in temperature from 700 to 800 °C resulted in a slight decrease in surface area, which may be attributed to the deformation of the structure of the prepared activated carbon.

**Table 1:** Analysis of the surface area of fabricated Nitrogen-doping activated carbons.

Analysis method	Sample entry		NDAC600	NDAC700	NDAC800
	AC yield	(%)	25.03	25.757	21.545
BET	a_s, BET_ (m^2^∕g)	*S*_BET_ (m^2^/g)	553.63	783.78	727.34
	V_m_ (cm^3^/g)	V_m_ (cm^3^ (STP)/g)	124.2	180.08	167.11
	Mean pore diameter	*D*_p_ (nm)	2.0584	2.0194	1.9668
	Volume of total pore	*V*_T_ (cm^3^/g)	0.2849	0.3957	0.3576
t-plot	a_1_–a_2_	*S*_mi_ (m^2^/g)	640.649	894.315	864.64
	V2	*V*_mi_ (cm^3^/g)	0.2441	0.3515	0.3193
	2t	(nm)	0.7573	0.7813	0.7341
MP	a_1_-a_2_	(m^2^ ∕g)	604.981	851.67	810.177
	V_p_	(cm^3^∕g)	0.2536	0.3626	0.3337
BJH ads	*V*_p_	(cm^3^/g)	0.0822	0.109	0.0862
	a_p_	(m^2^/g)	76.743	109.49	84.85
BJH des	V_p_	*V*_me_ (cm^3^/g)	0.0512	0.0609	0.0499
	a_p_	*S*_me_ (m^2^/g)	32.867	40.58	32.378

#### EDX analysis

**Figure 4:**
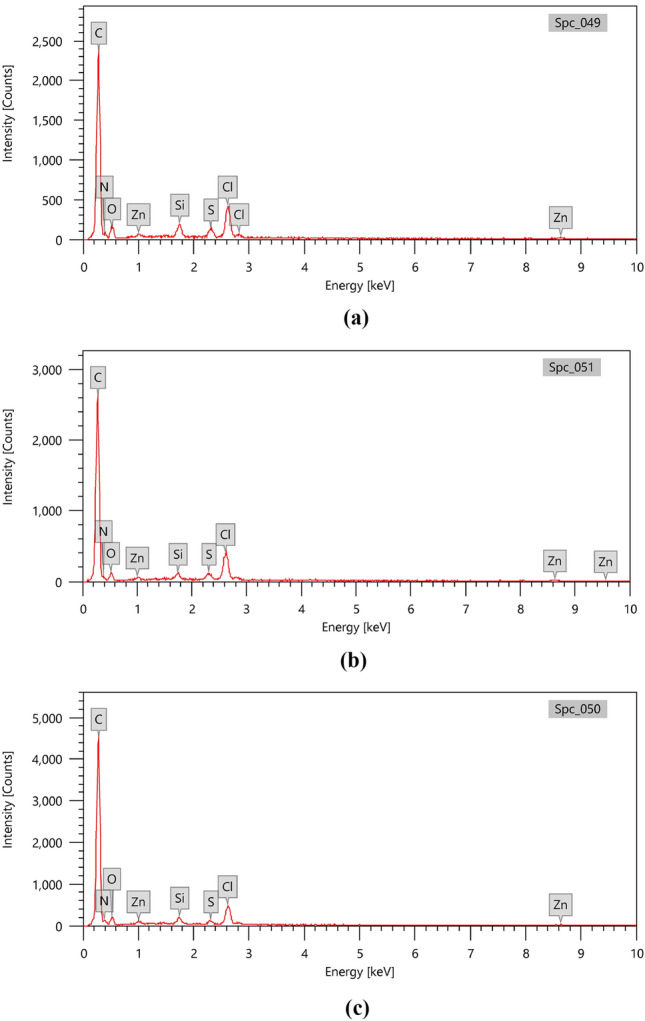
A EDX analysis of (**a**) NDAC600, (**b**) NDAC700, and (**c**) NDAC800.

About 60% of fish waste is a protein mainly containing C, N, O, H elements, and it is hydrolyzed at high temperatures. The process of preparing of self nitrogen-doped porous activated carbons (NDACs) by hydrothermal at 180 °C and then pyrolysis under nitrogen gas flow at high temperatures (600, 700, and 800 °C) is actually the removing method of non-carbon elements and carbon skeleton rearrangement with introducing of the nitrogen atom in the carbon network. Zinc chloride was used as an impregnation reagent to improve the pores formed during the pyrolysis process^58^. At higher temperatures, ZnCl_2_ will evaporate as a gas to form pores. ZnCl_2_ combines with oxygen in OH functional groups to cause a dehydrogenation and dehydration process of the hydroaromatic structure. Therefore, the NDAC600, NDAC700 and NDAC800, have higher carbon content but a relatively low oxygen as shown in Table 2. According to EDX analysis of NDAC600 possessed 74.19% of carbon, a high amount of nitrogen that was 8.13%, 11.18% oxygen, low amounts of silicon and sulphur, which were 0.94 and 0.86%, respectively, 3.79% chlorine and 0.9% zinc. The EDX analysis of NDAC700 consisted of 81.38% carbon, 4.21% nitrogen, 8.41% oxygen, low amounts of silicon and sulphur, which were 0.52 and 0.72%, respectively, 3.75% chlorine and 1.01% zinc. While the EDX analysis of NDAC800 consisted of 76.73% carbon, 9.85% nitrogen, 8.97% oxygen, low amounts of silicon and sulphur, which were 0.60 and 0.43%, respectively, 2.70% chlorine and 0.73% zinc (Table 2, Fig. 4a–c). From these results it can deduced that the NDAC800 has the highest nitrogen content.

**Table 2:** Element analysis of NDAC600, NDAC700, and NDAC800 using EDX analysis.

Element	NDAC600		NDAC700		NDAC800	
	Mass %	Atom %	Mass %	Atom %	Mass %	Atom %
C	74.19 ± 0.33	80.88 ± 0.36	81.38 ± 0.34	87.27 ± 0.37	76.73 ± 0.24	82.18 ± 0.26
N	8.13 ± 0.55	7.60 ± 0.52	4.21 ± 0.53	3.87 ± 0.49	9.85 ± 0.48	9.04 ± 0.44
O	11.18 ± 0.34	9.15 ± 0.28	8.41 ± 0.31	6.77 ± 0.25	8.97 ± 0.25	7.21 ± 0.20
Si	0.94 ± 0.04	0.44 ± 0.02	0.52 ± 0.04	0.24 ± 0.02	0.60 ± 0.03	0.27 ± 0.01
S	0.86 ± 0.04	0.35 ± 0.02	0.72 ± 0.04	0.29 ± 0.01	0.43 ± 0.02	0.17 ± 0.01
Cl	3.79 ± 0.08	1.40 ± 0.03	3.75 ± 0.08	1.36 ± 0.03	2.70 ± 0.05	0.98 ± 0.02
Zn	0.90 ± 0.10	0.18 ± 0.02	1.01 ± 0.11	0.20 ± 0.02	0.73 ± 0.07	0.14 ± 0.01
Total	100.00	100.00	100.00	100.00	100.00	100.00

#### Scanning electron microscope (SEM)

**Figure 5:**
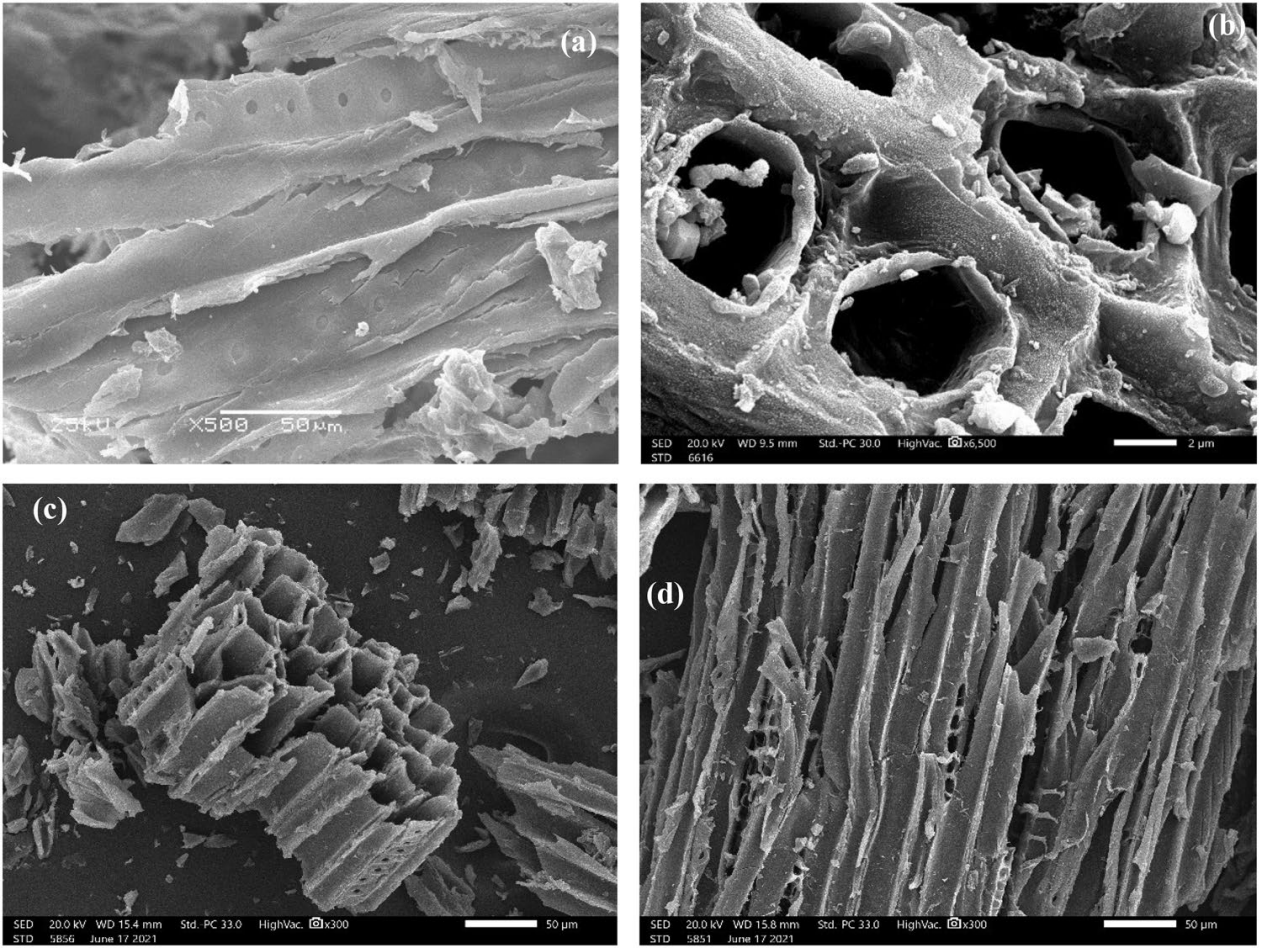
SEM analysis image of (**a**) raw sawdust, (**b**) NDAC800, (**c**) NDAC700, and (**d**) NDAC600.

Figure 5a–d displayed the SEM pictures of raw sawdust, NDAC800, NDAC700 and NDAC600, respectively. From Fig. 5a, the raw sawdust appears as a heterogeneous macroscopic smooth surface with large numbers of crumples and lappets. Also, Fig. 5b shows that the nitrogen-doped activated carbon prepared at 800 °C has obvious channel-pores with an abundant uniform distribution of channel-pores size. The average size of all micropores is < 2 nm as proved by BET analysis of the samples (Table 1). This result proved that the N-atoms were successfully introduced into the skeleton structure of activated carbons with the highest microporosity. Figure 5c and d represent the SEM images of NDAC700 and NDAC600, respectively.

#### X-Ray diffraction (XRD) study

**Figure 6:**
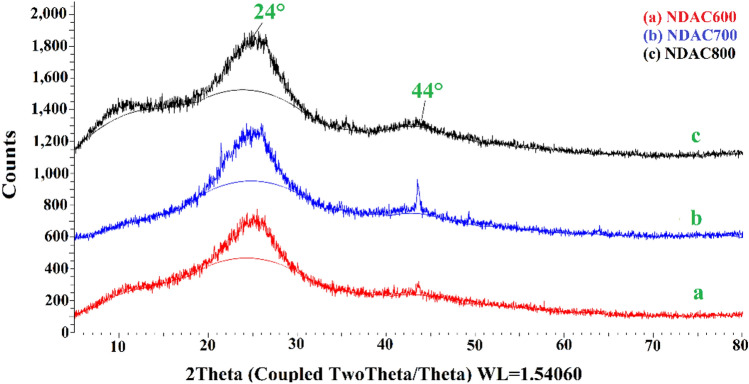
The XRD analyses of (**a**) NDAC600, (**b**) NDAC700 and (**c**) NDAC800.

XRD analysis of the fabricated NDAC600, NDAC700 and NDAC800 were presented in Fig. 6. The XRD spectra of all nitrogen-doped activated carbons reflect two peaks around 24° and 44° assigned to the (002) and (101) plans of carbons, respectively. In this case, the weak intensity peak would reveal the smaller crystallites, which is convenient with the amorphous structure of nitrogen-doped activated carbon.

#### X-ray Photoelectron Spectroscopy (XPS)

**Figure 7:**
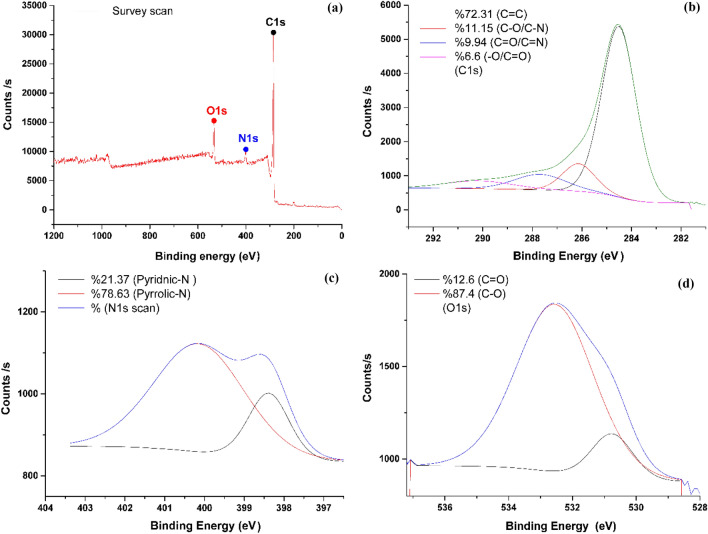
(**a**) Overview spectrum of NDAC800 with 1 eV resolution, (**b**) C1s, (**c**) N1s and (**d**) O1s High resolution of XPS core level spectra.

XPS was used to qualitatively analyze the functional groups on the surface of activated carbon^59,60^. Figure 7a is the wide full XPS spectra of precursor and NDAC800. As seen from the graphs, characteristic peaks of C1s, O1s, N1s are found in NDAC800, witnessing that N have been successfully retain on NDAC. The peaks located in 285.49, 400.33 and 533.05 eV are corresponding with C1s, N1s and O1s, respectively. Figure 7b C1s exhibits four peaks by curve fitting of the C1s spectrum. The C1s spectrum can be deconvoluted into four peaks centered at 284.51 (72.31%), 286.13 (11.15%), 287.68 eV (9.94%) and 290.18 eV (6.6%), assigned to sp^2^-C hybridized C=C bonds, C–O/C–N bonds, C=O/C=N bonds, and –O/C=O bonds respectively^61–63^. The N1s XPS spectra of NDAC800 could be de-convoluted into two types of N-containing compounds, and results are depicted in Fig. 7c. The peaks of N1s located in 398.38 (pyridinic N) and 400.14 (pyrrolic N)^63,64^, respectively in NDAC-600. Pyrrolic N is converted into two nitrogen-containing groups, namely 398.38 eV (pyridinic N), 400.14 eV and (pyrrolic N). The presence of pyridinic and pyrrolic N promotes the ion transport from the electrolyte to electrode material, effectively enhancing the capacitive properties. The O1s XPS spectrum of the NDAC-600 shown exhibits two peaks in Fig. 7d at 530.75 eV, and 532.54 eV, corresponding to (C=O) and (C–O).

#### Thermal analysis

**Figure 8:**
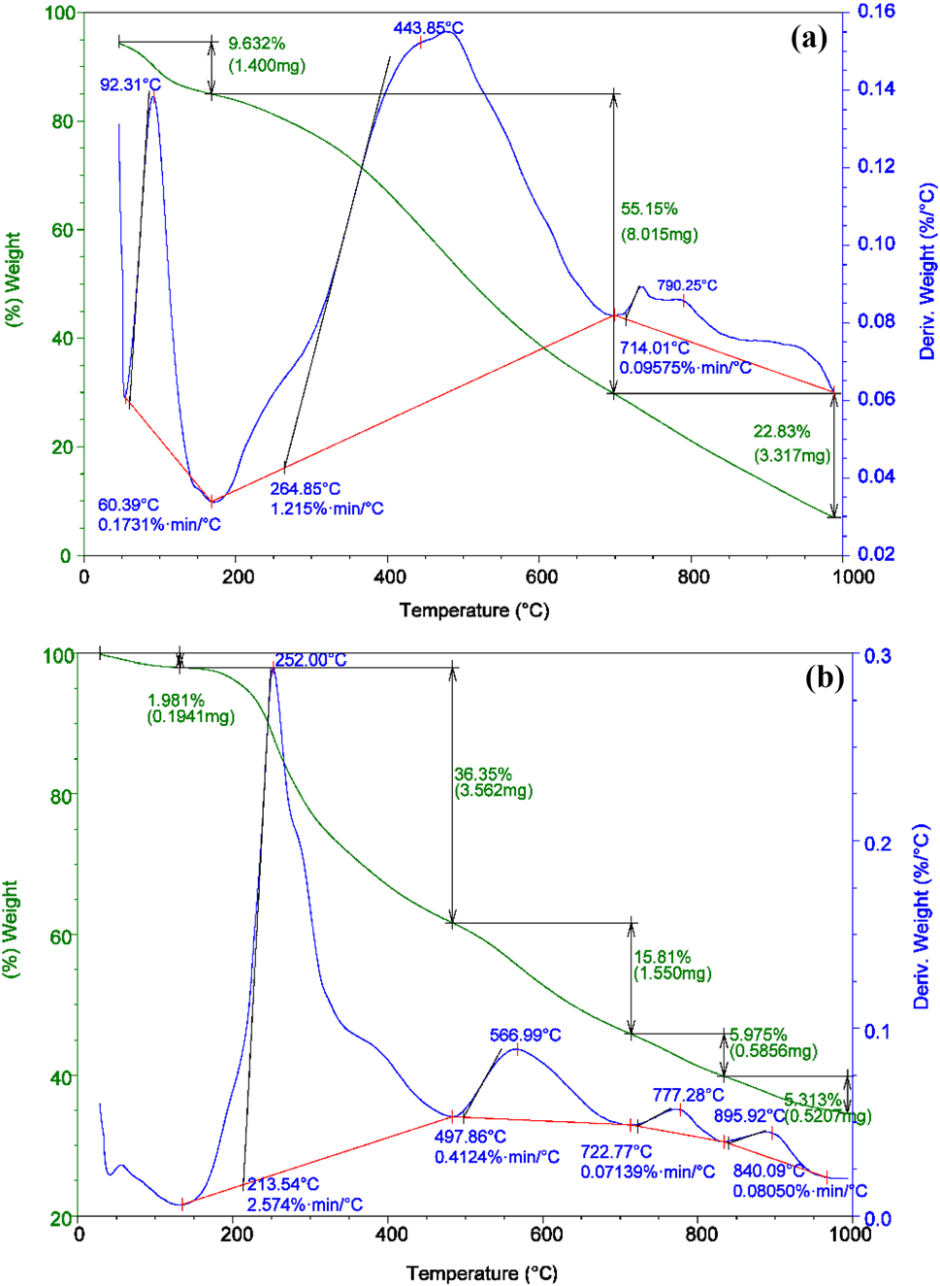
DTA and TGA analyses of (**a**) Sawdust, (**b**) Sawdust/fish waste/ZnCl_2_/urea (5:5:5:1) mixture.

The mixture of fish waste (60% protein content)/sawdust/zinc chloride/urea with mass ratio (5:5:5:1), subjected to a hydrothermal process at 180 °C for 5 h were analyzed for TGA and DTA. A multistage of thermochemical decomposition of sawdust was observed, as shown in Fig. 8a. The occurrence of the first weight loss of 9.63% in the temperature range of 60.39–190 °C can be attributed to the lignocellulosic material dehydration. Second mass-loss of 55.15% appears in the range between 190 and 714 °C, and the maximum mass loss occurred at 443.85 °C. The third weight mass loss of 22.83% appears in the temperature range of 714–980 °C and the maximum weight loss occurred at 790.25 °C which is may be due to the presence of unstable cellulosic fragments and the depolymerization of cellulose. The representative TGA and DTA curves of the mixture of the samples subjected to hydrothermal process at 180 °C for 5 h, is presented in the typical Fig. 8b. The TGA and DTA results indicate that, the major thermochemical decompositions take place in the temperature ranges of 25–145, 150–477, 497.86–720.77, 722.77–838.00 and 840.09–990.00 °C with weight loss 1.981, 36.35, 15.81, 5.975 and 5.313%, respectively. In the first stage, in the temperature range of 25–145 °C, it was observed that a small amount of weight loss shown in TGA curve is accompanied by a small peak in DTA curve corresponding to the release of moisture. The second stage showed a significant weight loss from 150 to 477 °C accompanied by a large peak shown in DTA curve. In the third stage, a moderate weight loss occurred within the temperature changed from 497.86 to 722.77 °C. While the fourth stage showed a little weight loss of 5.975% at temperature range of 722.77–840.09 °C which may be contributed to the release of ZnCl_2_ (melting point ~ 283 to 290 °C and boiling point of 732 °C) accompanied by a few volatiles organic compounds. The last stage showed a little weight loss of 5.313% in the temperature range between 840.09 and 990.00 °C.

**Figure 9:**
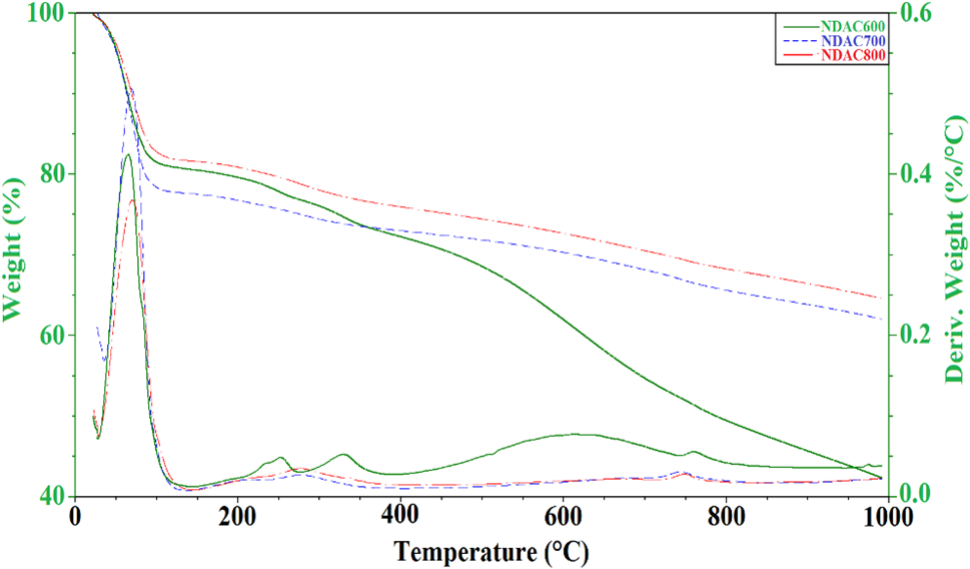
DTA and TGA analyses of NDAC600, NDAC700, and NDAC800.

Figure 9 reported the DTA and TGA analyses of the prepared NDAC600, NDAC700, and NDAC800. The first weight loss occurred for the three samples was 19.32, 22.30, and 18.27%, respectively in the temperature range of 25–150 °C which can be attributed to the water content in the samples. For the NDAC600, four more weight losses were occurred as 3.79, 4.29, 20.23, and 9.92% appears in the range between 150 and 1000 °C, and the maximum mass loss occurred at 400 to 750 °C (20.23%), which may be attributed to complete decomposition of the NDAC600. For the NDAC700 and NDAC800, almost similar two more weight losses were reported between 150 and 1000 °C, which proved that when the pyrolysis occurred at 700 and 800 °C, the produced NDACs were have almost similar thermal stability (Fig. 9). The DTA results supported the TGA analyses for the prepared NDAC600, NDAC700, and NDAC800. In the first stage, in the temperature range of 25–150 °C, it was observed that about 20% of weight loss shown in TGA curve is accompanied by strong peaks in DTA curve corresponding to the release of moisture. The remaining stage showed a significant weight loss from 150 to 1000 °C accompanied by a multiple small peaks shown in DTA curve (Fig. 9).

#### Testing of prepared NDACs for the adsorption of AY36 dye

**Figure 10:**
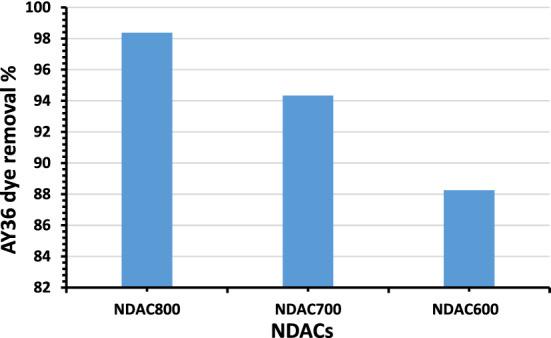
Removal of AY36 dye using NDACs prepared at different temperature under N_2_ gas flow.

All the prepared NDACs were tested for the removal of AY36 dye from water and the more efficient was selected for further studies. The obtained results were shown in Fig. 10. The NDAC800 sample has the highest removal rate (98.37%), while NDAC600 has the lowest removal rate (88.25). Therefore, the NDAC800 was selected to investigate the AY36 dye removal through this study.

### Adsorption studies of AY36 dye

#### pH on AY36 dye removal

**Figure 11:**
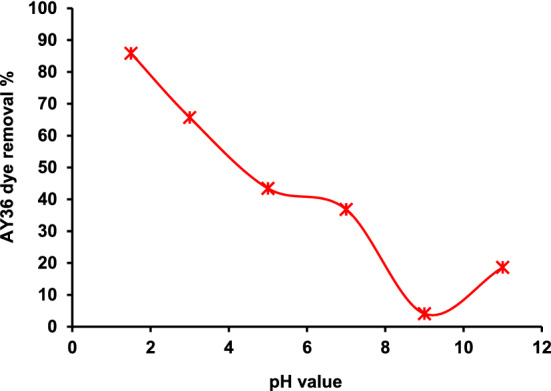
pH on the AY36 dye removal by NDAC800 (1.0 g/L) and 100 mg/L initial dye concentration at 25 ± 2 °C.

The pH of an aqueous solution is one of the most important factors in the adsorption of cationic and anionic dyes because it influences both the surface binding sites of the adsorbent and the ionization process of the dye molecule. For this purpose, different pH values ranging from 1 to 11were tested for the removal of the considered dye. During pH effect studies, the following parameters were kept constant (The initial AY36 dye concentrations of 100 mg/L, the NDAC800 dose of 1.0 g/L, 200 rpm agitation speed, and 25 °C temperature). During the tests, pH values were changed by adding a few drops of 0.1 M NaOH or HCl. Figure 11 shows the effect of the pH values on the removal of AY36 dye onto NDAC800. The amount of AY36 dye removal decreased with an increase in the pH value. When the pH value was changed from 1.5 to 9, the removal efficiency was decreased from 85.86 to 4.07%. While at pH = 11, the removal efficiency increased to 18.64%. The optimum pH was found to be 1.5 with the highest removal efficiency of 85.86%. The protonation state of the adsorbent may account for the higher removal efficiency of AY36 dye from NDAC800 at lower pH values^65,66^. Electrostatic repulsion between the anionic AY36 dye molecules and the negatively charged surface of NDAC800 was produced by the increase in pH value, which decreased the positively charged sites number and raised the negatively charged sites number^67–69^.

#### Contact time effect on AY36 dye removal

**Figure 12:**
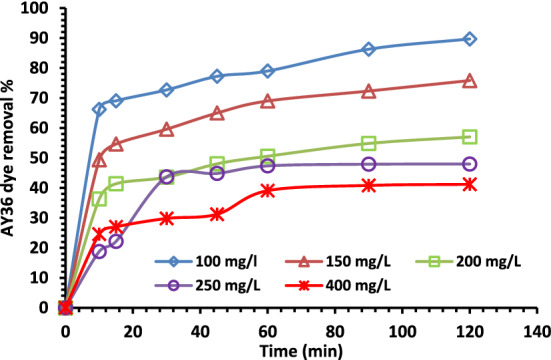
Effect of contact time on the removal rate of AY36 dye at different initial concentrations using 1.0 g/L of NDAC800 at 25 ± 2 °C.

All transfer phenomena, including the adsorption process, unavoidably involve contact time as a fundamental characteristic. Figure 12 shows the contact time of effect of the on the removal rates of AY36 dye on NDAC800 for 120 min. It is clearly shown in Fig. 12, the most of AY36 dye is removed after 10 min, and the removal percentage at 100 mg/L initial concentration of AY36 dye was 66.18% while the removal rate was 49.35% at the initial concentration 150 mg/L. After that, the removal rate tastily increased until it reached the equilibrium after 120 min and became stable at 89.707 and 75.82%, respectively. At the initial concentrations of 200, 250, and 400 mg/L, the removal rate after 10 min was 36.28, 18.79, and 24.55%, respectively, and increased slowly with marginal and moderate values until it reached the equilibrium after 120 min and become 57.10, 47.96 and 41.18%, respectively. It is proved that the amount of AY36 dye that was removed by NDAC800 grew rapidly at first (helping with external surface adsorption) and then progressively over a longer period of time until equilibrium was established (contributing to internal surface adsorption). This effect was noticed due to the high concentration of the solution and all the active sites on the NDAC800 surface were initially unoccupied. After that time, there weren't many surface active sites accessible, so the amount of AY36 dye removed increased extremely slowly^70^.

The AY36 dye was fast up taken onto NDAC800 to its large specific surface areas, adsorbent-adsorbate interaction, porous structures and pore volumes. This electrostatic attraction is due to the lone pair of electrons that are carried by nitrogen atoms of NDAC800 and delocalize the initial *sp*^*2*^ hybrid electron cloud on the carbon skeleton, increasing both surface reactivity and electron transport.

#### Initial concentrations effect and adsorbent dosage

**Figure 13:**
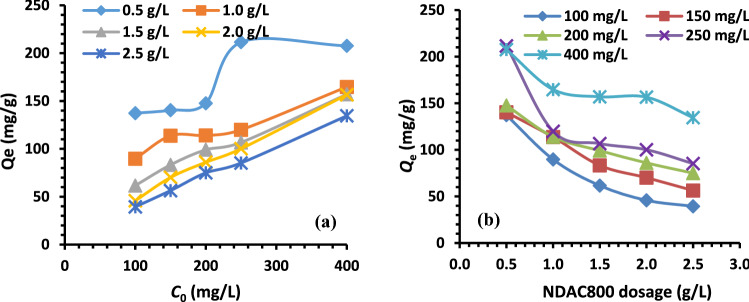
(**a**) Relation between *Q*_e_ (mg/g) and different initial concentrations of AY36 dye (100–400 mg/L) using different NDAC800 dosages (0.5–2.5 g/L); (**b**) plots of *Q*_e_ against different NDAC800 dosages (0.5–2.5 g/L) using different initial dye concentrations (100–400 mg/L) at 25 ± 2 °C.

The *initial concentrations effect* of AY36 dye solutions on the removal rate was investigated. An experimental contact time of 120 min and a pH of 1.5 were chosen (determined previously) to study the effect of initial concentration (100, 150, 200, 250, and 400 mg/L) and NDAC800 dosages (0.50–2.50 g/L). Figure 13a displays the plot of *Q*_e_ (mg/g) versus initial concentration. At the adsorbent dose of 0.50 g/L and the initial dye concentration ranging from 100 to 400 mg/L, the equilibrium uptake capacity, *Q*_e_ (mg/g), increased from 137.263 to 207.639 mg/g. At the adsorbent doses of dye (1.00, 1.50, 2.00 and 2.50 g/L) and the initial dye concentration of 100–400 mg/L, the equilibrium uptake capacity, *Q*_e_ (mg/g) increased from 89.707 to 164.736, 61.625 to 156.970, 45.834 to 156.588 and 39.412 to 134.513 mg/g, respectively. It is observed that, the dye maximum adsorption capacity decreased from 207.639 to 134.513 mg/g due to increasing in NDAC800 adsorbent dosage from 0.5 to 2.5 g/L and at intial dye concentration of 400 mg/L. Figure 13b shows the plot of *Q*_e_ (mg/g) versus various NDAC800 doses (0.50–2.50 g/L) using different initial dye concentrations of (100–400 mg/L). At NDAC800 dose 0.5 g/L and the initial dye concentration of 100 mg/L, the *Q*_e_ decreased from 137.263 to 39.412 mg/g. At absorbent doses 1.00, 1.50, 2.00 and 2.50 g/L of NDAC800 and the initial concentration of AY36 dye 150, 200, 250, and 400 mg /L, the *Q*_e_ decreased from 140.357 to 56.359, 147.652 to 74.987, 211.522 to 85.240 and 207.639 to 134.513 mg/g, respectively. From the results, it can be deduced that the equilibrium uptake capacity (*Q*_e_) at initial concentration ranging from 100 to 250 mg/L increases from 137.263 to 211.522 mg/g, while at initial dye concentration 400 mg/L decreased to 207.639 mg/g for an increase in the AY36 dye concentration from 100 to 400 mg/L using NDAC800 dose 0.5 g/L. *Q*_e_ at 250 mg/L as dye initial concentration and NDAC800 dosage 0.5 g/L possessed the highest value, 211.522 mg/g. Because more vacant adsorption sites are available due to rising the amount of NDAC, more dye may be absorbed, resulting in a rise in dye removal percentage.

The increase in NDAC800 dose causes a decrease in the amount of AY36 dye adsorbed onto the unit weight of NDAC800, which results in a lower solute concentration, and this decrease in AY36 dye adsorbed onto NDAC800 dose weight may be the cause of the decrease in *Q*_e_ value (concentration gradient or splitting effect) between sorbets and sorbent. The initial concentration gradient between the solid adsorbent and the bulk liquid can be used to explain this. The driving force, or initial AY36 dye concentration incline, is likely to cause a rise in the amount of dye absorbed per unit weight of adsorbent. However, in this instance, the initial concentration incline between the adsorption vacant sites of NDAC800 and the concentration of AY36 dye solutions gets smaller with increasing adsorbent mass, directing to a decrease in *q*_e_ value for a fixed volume of dye solution and initial dye concentration. It can be seen from Fig. 13a and b that for all of the adsorbent masses examined, the concentration in AY36 dye solution was greater than the maximum number of unoccupied sites that could be found at the surface of the adsorbent. But it was nevertheless discovered that the *Q*_e_ value fell as NDAC800 mass rose. This impact may be ascribed to a decrease in the adsorbent’s overall surface area, which is likely the result of aggregation during the adsorption process^71,72^.

### AY36 dye adsorption isotherms models

Isotherm of adsorption, a useful tool, often describes the mechanisms controlling the retention or mobility of material from aqueous porous media or aquatic habitats to a solid phase at a constant conditions^73,74^. Once an adsorbate-containing phase has been in contact with the adsorbent for enough time, its adsorbate concentration in the bulk solution becomes in a dynamic balance with the interface concentration; equilibrium of the adsorption (the ratio between the amount of pollutant adsorbed and the amount remaining in the solution) is established^75,76^. The mathematical relationship, which is frequently used and shown by expressing the solid phase versus its remaining liquid concentration^77^, typically plays an important role in the modelling analysis, working design, and practical application of adsorption methods. Understanding the adsorption mechanism, surface properties, and degree of adsorbent affinities is possible due to the parameters of this substance's physicochemistry and the underlying thermodynamic theories^78^.

Numerous adsorption isotherm models, namely Langmuir (LIM), Freundlich (FIM), Dubinin-Radushkevich (DRIM), Temkin (TIM), and Halsey isotherm (HIM), have been developed over time to analyse experimental data^78–80^. The first method mentioned is kinetic consideration.

While thermodynamics, the base of the second approach, can give an outline for generating several types of adsorption isotherm models^81,82^ and potential theory. The third approach, often transmits the primary notion in the production of the distinguishing curve^83^. However, the derivation in more than one way, which points to the discrepancy in the physical interpretation of the model parameters, is an intriguing development in isotherm modeling. The experimental results in this study were investigated using the above mentioned equilibrium isotherm models^78^.

#### Langmuir isotherm model (LIM)

The LIM^80^ undertakes uniform energies of removal of a solute from a fluid solution onto a surface comprising a definite number of identical sites as a monolayer adsorption with no adsorbate transmigration into the surface plane^84^. Therefore, LIM was selected to estimate the maximum adsorption capacity (*Q*_m_, mg/g) of complete monolayer coverage on the surface of sorbent. LIM is expressed by Eq. (4) (Table 3), where *q*_e_ (mg/g) is the mono-layer adsorption capacity of adsorbent at equilibrium and *K*_L_ (L/mg) is the LIM adsorption constant. Therefore, a plot of *C*_e_/*q*_e_ versus *C*_e_ gives a straight line of slope 1/*Q*_m_ and intercepts 1/(*Q*_m_*K*_L_). The LIM plot is shown Fig. 2Sa in supplementary material, and the LIM data are given in Table 4.

**Table 3:** The equations of isotherm models (IM).

Isotherm models	Linear equation	Equation no.
Langmuir	$\frac{{C}_{e}}{{q}_{e}}=\frac{1}{{K}_{L}{Q}_{m}}+\frac{1}{{Q}_{m}}{C}_{e}$	(4)
Freundlich	$\mathrm{log}{q}_{e}=\mathrm{log}{K}_{F}+\frac{1}{n}\mathrm{log}{C}_{e}$	(5)
Temkin	${q}_{e}=\frac{RT}{B}\mathrm{ln}A+\frac{RT}{B}ln{C}_{e}$	(6)
Dubinin-Radushkevich	$\mathrm{ln}{q}_{e}=\mathrm{ln}{q}_{m}-\beta {\varepsilon }^{2}$	(7)
Halsey	$ln{q}_{e}=\frac{1}{n}\mathrm{ln}k+\frac{1}{n}\mathrm{ln}{C}_{e}$	(8)

**Table 4:** Isotherm models investigation data of AY36 dye adsorption of 100–400 mg/L as initial dye concentration onto 0.5–2.5 g/L NDAC800 doses at 25 ± 2 °C.

Isotherm models	IM parameters	NDAC800 (g/L)				
		0.5	1.0	1.5	2.0	2.5
Langmuir	*Q*_*m*_ (mg/g)	232.56	169.49	169.49	188.68	142.86
	*K*_*a*_ × 10^3^	25.34	38.89	35.04	36.53	86.85
	*R*^*2*^	0.947	0.948	0.932	0.892	0.897
Freundlich	*1/n*	0.21	0.16	0.28	0.42	0.30
	*K*_*F*_ (mg^1-1/n^L^1/n^/g)	63.61	60.62	33.88	21.90	32.94
	*R*^*2*^	0.619	0.815	0.946	0.895	0.921
Temkin	*A*_*T*_	1.19	7.89	0.93	0.45	2.53
	*B*_*T*_	34.93	19.40	27.59	37.91	22.29
	*R*^*2*^	0.600	0.748	0.861	0.863	0.810
Dubinin-Radushkevich	*Q*_*m*_ (mol/kg)	178.70	128.09	111.37	117.08	84.88
	*K* × 10^6^ (mol/kJ)^2^	48,800	6900	6500	10,900	500
	*E* (kJ/mol)	3.20	8.51	8.77	6.77	31.62
	*R*^*2*^	0.327	0.537	0.609	0.780	0.565
Halsey isotherm	*1/n*_*H*_	0.2053	0.1611	0.2796	0.4197	0.3025
	*K*_*H*_	6.09E+08	1.16E+11	2.96E+05	1.56E+03	1.04E+05
	*R*^*2*^	0.6189	0.8148	0.9458	0.8895	0.9208

#### Freundlich isotherm model (FIM)

The FIM is the initially reported equation investigating the adsorption process^85^. The FIM expression assumes that because it is an exponential equation, the concentration of adsorbate on the adsorbent surface increases as well as the adsorbate concentration does. The FIM can be expressed as Eq. (5) (Table 3), where *K*_F_ and *n* are the FIM adsorption constants, which can be measured by the linear plot of log *q*_e_ versus log *C*_e_. *1/n* is a constant revealing of the intensity of the adsorption of dye onto the surface heterogeneity sorbent, becoming more heterogeneous as its value gets closer to zero. A value of *1/n* below one indicates a normal LIM, while *1/n* above one is revealing cooperative adsorption. The FIM plot is presented in Fig. 2Sb in supplementary material, and the FIM parameters are given in Table 4.

#### Temkin isotherm model (TIM)

The TIM made the assumption that all of the molecules in the layer's heat of adsorption decreased linearly with coverage as a result of interactions between adsorbents and adsorbates and that the adsorption was characterized by a uniform distribution of binding energies up to a maximum binding energy^86,87^. TIM is expressed as Eq. (6), where A and B are TIM constants, *R* is the gas constant, and *T* is the absolute temperature. The applicability of the isotherm was confirmed by a linear plot of *q*_e_ versus ln *C*_e_, which can be used to determine the constants A and B as shown in Fig. 2Sc in supplementary material and reported in Table 4.

#### Dubinin-Radushkevich model (DRIM)

The DRIM^88,89^ assesses the apparent porosity free energy and adsorption characteristics. The dose of DRIM not undertakes a constant sorption potential or homogeneous surface. The DRIM can be written as Eq. (7) (Table 3)^90^, where *β* (mmol^2^/J^2^) is a coefficient corresponding to the adsorption mean free energy of, *q*_m_ (mmol/g) is the maximum adsorption capacity and *ε* (J/mmol) is the polanyi potential which is written as: *ε* = *RT*(1 + 1/*C*_e_). The DRIM plot is shown in Fig. 2Sd in supplementary material, and the DRIM parameters are given in Table 4.

#### Halsey model (HIM)

Multilayer adsorption, and heteroporous substances may fit the HIM^91,92^. HIM is expressed as Eq. (8) (Table 3), where *K* and *n* are the HIM adsorption constants that can be obtained from the linear plot of ln *q*_e_ versus ln *C*_e_ as shown in Fig. 2Se in supplementary material and the obtained data were included in Table 4.

#### Error functions investigation (EFI)

In recent years, there have seen a rise in the use of linear regression, which is the most effective method for determining the best fitting^78,93^, mathematically analyzing adsorption systems, measuring the distribution of adsorbates^94^, and confirming the reliability and theoretical premises of an isotherm model^95^.

Because of the characteristic bias caused by the transformation in this study, which rides towards a variety of parameter approximation errors and fits distortion, a number of mathematically rigorous error functions (including the hybrid function fractional error (HYBRID), ARE (average relative error), *Χ*^2^ (nonlinear chi-square test), MPSD (Marquardt's percent standard deviation), ERRSQ (sum square error), EABS (sum of absolute errors), APE (average percentage errors), and root mean square error) have done. Based on its convergence criteria, nonlinear regression often includes either minimization or maximization of the error distribution (between the experimental data and the predicted isotherm)^96^. This is in contrast to linearization models. Despite the fact that the Sum Square Error (ERRSQ) is the most frequently employed error function^78,97^, the magnitude and squares of the errors tend to grow at the liquid-phase higher-end concentration ranges, demonstrating a better fit for parameters derivation of the isotherm^98^. Equation (9) represents ERRSQ (Table 5).

**Table 5:** Error functions models applied to investigate of the various isotherm models applicability.

EM name	Error equation	Equation no.
ERRSQ	$ERRSQ= \sum_{i=1}^{n}{\left({q}_{e,isotherm}-{q}_{e,cal}\right)}^{2}$	(9)
HYBRID	$Hybrid= \frac{100}{p-n} \sum_{i=1}^{p}\left(\frac{{\left({q}_{e, isotherm}-{q}_{e, cal}\right)}^{2}}{{q}_{e,isotherm}}\right)i$	(10)
ARE	$ARE= \frac{100}{n}+\sum_{i=1}^{n}\left|\frac{{q}_{e,isotherm}-{q}_{e,cal}}{{q}_{e,isotherm}}\right|i$	(11)
EABS	$EABS= \sum_{i=1}^{n}\left|{q}_{e,isotherm}-{q}_{e,cal}\right|i$	(12)
MPSD	$MPSD=100\times \sqrt{\frac{\sum_{i=1}^{n}{\left(\frac{{q}_{e, isotherm}-{q}_{e, cal}}{{q}_{e,isotherm}}\right)}^{2}}{n-p}}$	(13)
X^2^	${X}^{2}= \sum_{i=1}^{n}\left(\frac{\left(qemeas-qecal\right)^2}{qeexp}\right)$	(14)
APE	$APE\%= \frac{100}{n}\times \sum_{i=1}^{n}\left|\frac{{q}_{e,isotherm}-{q}_{e,cal}}{{q}_{e,isotherm}}\right|i$	(15)
RMS	$RMS=100\times \sqrt{\frac{\sum_{i=1}^{n}{\left(1-\frac{{q}_{e, cal}}{{q}_{e, isotherm}}\right)}^{2}}{n}}$	(16)

The development of the error function improved the HYBRID fit at low concentrations. Here, each value of ERRSQ is divided by the solid-phase concentration of the experimental with a term for the degrees number of freedom (the data point number minus the parameters number in the isotherm equation) built into the system^99^. ARE model^100^ shows a propensity to underestimate or overestimate the experimental data, and aims to reduce the fractional error distribution over the whole concentration range under study. The ERRSQ function and the sum of absolute errors (EABS) both use an approach where an increase in errors will result in a better fit and a bias toward data with high concentrations^101^. A number of scholars have previously used MPSD (Eq. 13) error function^102^ in the isotherm investigations^103–106^. It resembles a modified geometric mean error distribution in some ways, depending on the system's degree of freedom^100^. The nonlinear chi-square test, which can be calculated by comparing the sum squared differences between calculated and experimental data and dividing each squared difference by its corresponding value, is a statistical tool required for the best-fit of an adsorption system (*Χ*^2^ is expressed as Eq. (14). While a higher number reflects the variance of the experimental data, a smaller X^2^ value indicates its similarities^95^ .According to Eq. (15), the average percentage errors (APE) show how well the observed and anticipated values of adsorption capacity used to create isotherm curves match together^99^. Equation (16) gives the root mean square errors (RMS)^99^ (Table 5).

The *q*_e_ (211.552 mg/g) and the *Q*_m_ (232.56 mg/g) are in excellent agreement, and an evaluation of the isotherms based on their coefficient of determination (*R*^2^) (0.948) revealed that the LIM is the best model fits the data (Table 6). However, when comparing the isotherms based on error functions, Temkin has the least error functions, roughly equal. However, the adsorption of the AY36 dye onto NDAC800 could be adequately predicted by the two isotherm models, according to the data reported above.

**Table 6:** The isotherm models Error function analysis of the AY36 dye adsorption of different *C*_0_ (100–400 mg/L) onto NDAC800 different doses (0.5–2.5 g/L) at 25 ± 2 °C.

IM name	APE%	X^2^	ERRSQ	Hybrid	MPSD	ARE	EABS	RMS
Langmuir	0.10	1.90	213.30	8.27	0.54	0.10	73.02	0.52
Freundlich	0.03	0.15	16.35	0.63	0.15	0.03	20.22	0.14
Halsey	10.45	5257.84	162,027.72	22,860.18	54.47	10.45	2012.63	52.25
Temkin	0.00	0.00	0.00	0.00	0.00	0.00	0.02	0.00
D-R	0.08	1.11	124.66	4.83	0.42	0.08	55.83	0.40

### AY36 dye adsorption kinetic models

The rate controlling the process of adsorption was studied using a number of kinetic models, including mass transfer, diffusion control, and chemical reaction. The kinetics of AY36 dye adsorption onto NDAC800 was investigated in order to choose the best working conditions for a full-scale batch process because the parameters of kinetic are important for the adsorption rate prediction and provide crucial evidence for scheming and modeling the adsorption processes. Consequently, pseudo-first order (PFOM)^107,108^, pseudo-second-order (PSOM)^109^, Elovich (EM)^110–112^, intraparticle diffusion (IDM) and film diffusion (FDM) kinetic models^113,114^ were used for AY36 dye adsorption onto NDAC800 (Table 7). The conventionality between the model-predicted values and experimental data was expressed by the *R*^2^ (correlation coefficients), as shown in Fig. 3Sa–e in supplementary material and Tables 8, 9.

**Table 7:** The used adsorption kinetic models equations.

Model name	Model Equation	Equation no.
PFO	$\mathrm{log}\left({q}_{e}-{q}_{t}\right)=\mathrm{log}{q}_{e}-\frac{{K}_{1}}{2.303}t$	(17)
PSO	$\frac{t}{{q}_{t}}=\frac{1}{{K}_{2}{q}_{e}^{2}}+\frac{t}{{q}_{e}}$	(18)
Elovich kinetic	${q}_{t}=\frac{1}{\beta }\mathit{ln}\left(\propto \beta \right)+\frac{1}{\beta }ln(t)$	(19)
Intraparticle diffusion	${q}_{t}={K}_{dif}{t}^{0.5}+C$	(20)
Film diffusion	$\mathit{ln}\left(1-F\right)={K}_{FD}(t)$	(21)

**Table 8:** PFOM and PSOM rate constants as well as the experimental and calculated *q*_e_ values of AY36 dye adsorption of 100–400 mg/L as *C*_0_ and 0.5–2.5 g/L NDAC800 doses at 25 ± 2 °C.

Parameter			PFOM			PSOM		
NDAC800 (g/L)	AY36 (mg/L)	*q*_*e*_ (exp.)	*q*_*e*_ (calc.)	*k*_*1*_ × 10^3^	*R*^*2*^	*q*_*e*_ (calc.)	*k*_*2*_ × 10^3^	*R*^*2*^
0.5	100	137.26	81.55	30.86	0.977	149.25	0.601	0.998
	150	140.36	64.48	33.85	0.904	147.06	0.978	0.996
	200	147.65	125.46	38.46	0.961	166.67	0.388	0.994
	250	211.52	372.48	57.34	0.874	294.12	0.087	0.949
	400	207.64	252.70	27.64	0.798	303.03	0.052	0.796
1.0	100	89.71	31.98	22.57	0.946	92.59	1.551	0.996
	150	113.73	49.33	25.10	0.996	119.05	1.008	0.998
	200	114.02	54.80	26.71	0.976	120.48	0.902	0.997
	250	119.90	172.86	78.30	0.985	140.85	0.454	0.982
	400	164.74	153.53	47.90	0.908	181.82	0.442	0.989
1.5	100	61.63	20.34	39.38	0.861	63.69	3.567	0.998
	150	83.29	26.01	17.96	0.765	85.47	1.667	0.994
	200	99.12	42.70	22.11	0.983	104.17	1.054	0.998
	250	106.68	71.61	24.41	0.829	119.05	0.456	0.974
	400	156.97	117.14	31.78	0.914	175.44	0.371	0.988
2.0	100	45.83	0.54	17.04	0.277	44.64	94.672	0.999
	150	70.13	20.94	39.84	0.860	70.92	4.873	0.999
	200	86.00	32.67	22.11	0.902	90.09	1.341	0.994
	250	100.14	53.27	31.32	0.978	107.53	0.990	0.999
	400	156.59	65.45	32.24	0.891	163.93	0.926	0.996
2.5	100	39.41	2.99	22.11	0.894	39.68	20.033	1.000
	150	56.36	5.79	35.24	0.257	55.87	16.101	0.999
	200	74.99	30.01	24.87	0.962	78.13	1.682	0.997
	250	85.24	30.08	21.42	0.877	88.50	1.590	0.999
	400	134.51	67.20	34.31	0.819	140.85	0.923	0.994

**Table 9:** EM, IPDM, and FDM results of AY36 dye adsorption of 100–400 mg/L beginning concentrations onto 0.5–2.5 g/L NDAC800 doses at 25 ± 2 °C.

NDAC800 (g/L)	AY36 (mg/L)	EM			IPDM			FDM		
		*β*	*α*	*R*^*2*^	*K*_*dif*_	*C*	*R*^*2*^	*K*_*FD*_	*C*	*R*^*2*^
0.5	100	0.04	6.67E+01	0.987	7.49	60.82	0.956	0.03	0.52	0.977
	150	0.07	1.15E+03	0.938	4.95	88.38	0.962	0.03	0.78	0.904
	200	0.03	3.24E+01	0.963	9.81	48.78	0.933	0.04	0.16	0.961
	250	0.015	1.59E+01	0.969	20.88	9.12	0.847	0.00	0.92	0.000
	400	0.02	1.17E+01	0.848	19.90	13.55	0.921	0.03	0.20	0.798
1.0	100	0.11	9.83E+02	0.956	3.02	56.69	0.994	0.02	1.03	0.946
	150	0.06	1.74E+02	0.994	4.99	61.74	0.972	0.03	0.84	0.996
	200	0.06	1.48E+02	0.979	5.15	59.91	0.975	0.03	0.73	0.975
	250	0.03	1.71E+01	0.846	9.46	33.29	0.716	0.08	0.37	0.985
	400	0.03	7.54E+01	0.919	9.28	70.66	0.924	0.05	0.07	0.908
1.5	100	0.13	3.18E+02	0.772	2.26	40.25	0.685	0.02	2.26	0.254
	150	0.11	4.82E+02	0.843	2.99	50.76	0.844	0.02	1.16	0.765
	200	0.07	9.86E+01	0.995	4.63	50.45	0.963	0.02	0.84	0.983
	250	0.05	2.15E+01	0.860	6.88	32.66	0.844	0.02	0.40	0.829
	400	0.03	5.20E+01	0.941	9.31	58.67	0.974	0.03	0.29	0.914
2.0	100	− 1.81	1.29E−37	0.249	-0.15	45.68	0.180	0.01	2.69	0.026
	150	0.15	2.77E+03	0.884	1.97	50.65	0.767	0.01	2.39	0.012
	200	0.10	2.69E+02	0.846	3.40	49.54	0.865	0.02	0.97	0.902
	250	0.06	6.71E+01	0.961	5.09	48.85	0.898	0.03	0.63	0.978
	400	0.07	3.00E+03	0.904	5.04	102.92	0.953	0.03	0.87	0.891
2.5	100	1.24	9.00E+18	0.885	0.27	36.40	0.966	0.02	2.58	0.894
	150	0.26	1.20E+05	0.625	1.05	46.11	0.467	0.03	1.73	0.142
	200	0.11	2.83E+02	0.959	2.88	44.38	0.962	0.02	0.92	0.962
	250	0.09	1.63E+02	0.973	3.55	48.36	0.911	0.02	1.04	0.873
	400	0.07	1.33E+03	0.883	4.68	83.84	0.954	0.03	0.69	0.819

#### Pseudo-first-order model (PFOM)

The adsorption rate constant was measured by the Lagergren PFOM^108^, which is the initial known model describing the adsorption rate based on the adsorption capacity. The pseudo-first-order (PFO) kinetic model is shown by Eq. (17) as *q*_t_ and *q*_e_ (mg/g) are the quantities of ion adsorbed at time *t* and equilibrium, respectively, and *k*_1_ (min^−1^) is the PFO rate constant of adsorption process. The slope and intercept of the plots of log (*q*_e_ − *q*_t_) versus *t* were used to calculate the *k*_1_ and *q*_e_ (Fig. 3Sa in supplementary material).

#### Pseudo-second-order model (PSOM)

According to PSOM, the number of accessible sites on the adsorbent directly relates to the rate of solute adsorption. Additionally, the amount of solute on the adsorbent's surface affects the reaction rate. The *q*_e_–*q*_t_ (driving force) is relational to the active sites number available on the adsorbent^115,116^. The PSOM is shown by Eq. (18), where *k*_2_ (g/mg min) is the equilibrium rate constant of PSOM adsorption^117^. If the PSOM is appropriate, the plot of *t*/*q*_t_ versus *t* shows a linear relationship, and the *k*_2_ and *q*_e_ can be obtained from the slope and intercept of the line, respectively (Fig. 3Sb in supplementary material).

#### Elovich model (EM)

The EM aids in predicting a system's activation and deactivation energy as well as mass and surface diffusion. The model has been meaningfully applied to wastewater processing despite its initial application in gaseous systems. The model posits that as the amount of deposited solute increases, the rate of solute adsorption reduces exponentially^118,119^. EM undertakes the presence of heterogeneous active sites on adsorbent material^120^. EM is presented by Eq. (19), where *α* (mg/g min) and *β* (g/mg) are the beginning sorption rate constant and the surface coverage and activation energy for chemisorption, respectively. These constants can be calculated from the slope and intercept of *q*_t_ versus ln *t* plot following the model equation (Fig. 3Sc in supplementary material).

#### Intra-particle diffusion model (IPDM)

It is reasonable to suppose that the mechanism of adsorption for any dye removal using a solid phase material involves the subsequent four steps: (1) the transfer of color from a solution to the top of a solid phase (bulk diffusion); (2) the passage of dye through the boundary layer from the surface of the solid phase (FD); (3) the transfer of dye between the solid phase's surface and its particles' inner pores (intraparticle (IPD) or pore diffusion (PD)); and (4) the dye's adsorption at an active site on the surface of the solid phase (Chemical reaction such as ion-exchange, complexation and chelation). Usually, the mass transfer rates between the liquid phase and the IPD control dye adsorption^121^.

IPD may be the rate-regulating step in an experiment that uses a batch method and rapid stirring^122^. The IPDM was also investigated using Eq. (20)^123^, where *k*_dif_ is the IPD rate constant (mg/g min^1/2^) (Fig. 3Sd in supplementary material).

#### Film diffusion model (FDM)

Film diffusion (FD) is the adsorbate diffusion across the liquid film surrounding the particle of the adsorbent. FDM is expressed by Eq. (21), where *K*_FD_ is the external film mass transfer coefficient and *F* = *q*_t_/*q*_e_. The constant *K*_FD_ can be calculated from the slope and intercept of the ln (1−*F*) versus plot (Fig. 3Se in supplementary material)^124^.

The best fit kinetic model for the AY36 dye adsorption onto NDAC800 was the pseudo-second-order rate because PSOM rate results gave the highest determination coefficient (*R*^2^ = 1). According to the LIM and TIM adsorption isotherm models and the PSOM, it can be suggested that the first step involved the electrostatic attraction between the ions of hydrogen in the bulk solution and the equivalent negatively active sites on the self-doping activated carbon. This possessed numerous numbers of lone pairs of electrons of nitrogen atoms, then the AY36 dye adsorbed onto the surface of the positively self-doping activated carbon, and it formed a detectable adsorption monolayer. This first monolayer is most often formed for large species like dye molecules (AY36 dye), which cannot be penetrated through the micropores (mean pore diameter 1.9668 nm) according to specific surface area study and strong chemisorption onto a surface of the NDAC800 may be occurred. During the adsorption process, the adsorption heat varies with coverage of the amount of AY36 dye due adsorbate interactions, and then, the rat limiting step may be chemisorption involved through electrons involvement or exchange between the Acid Yellow 36 dye and the positively self-doping activated carbon.

### Compatrison results of Q_m_ of AY36 dye compared to those found in literature

The *Q*_m_ of AY36 dye removal using various adsorbents summarised in the literature were compared to the NDAC600 adsorbent (Table 10). This demonstrated that NDAC800 was effective for the adsorption of AY36 dye. The NDAC800 shows *Q*_m_ (232.56 mg/g), which is comparable to those reported in Table 10 for various adsorbent for the AY36 dye removal from water. İt was noticed that the NDAC800 was much more effective in relation to other adsorbents used for the AY36 dye removal.

**Table 10:** Comparison of the AY36 dye removal by NDAC800 with various adsorbents.

Precursor	*Q*_m_ (mg/g)	References
NDAC800	232.56	This study
Green nanoceria (GN) and GN–NH_2_ (AGN) from *Prosopis juliflora* leaves extract	GN (16.39) AGN (26.95)	^31^
Superabsorbent hydrogel of Poly(3-acrylamidopropyl)-trimethylammonium chloride-co-*N*,*N*-DMA	199.96	^125^
AC from peanut shells	66.70	^126^
Refused tea waste activated carbon	71.97	^127^
Date Seed as a biosorbent	22.07	^128^

**Figure 14:**
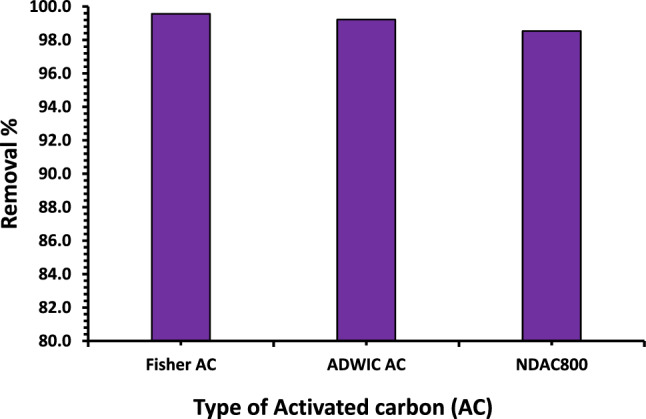
Removal % of AY36 dye using Fisher AC, ADWIC AC and NDAC800 using 2.5 g/L as adsorbent dose and 100 mg/L as initial dye concentration at 25 °C for 120 min.

Two commercial activated carbons (Fisher Scientific UK Charcoal activated code: C/4040/60 and ADWIC Egypt Charcoal activated powder) were tested in removing of AY36 dye ion from water compared with prepared NDAC800 (Fig. 14). The maximum removal % of AY36 dye obtained by the commercial activated carbons was ~ 99.56 and 99.22%, which are very closed to that obtained by NDAC800 (98.53).

### Regeneration of NDAC800

**Figure 15:**
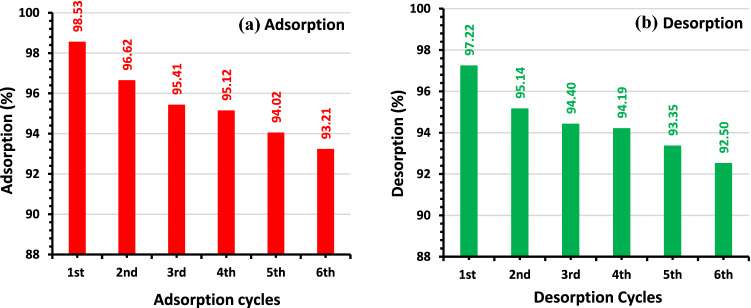
The effect of the regeneration rounds (cycles) (**a**) adsorption percentage of AY36 dye ion by NDAC800 and (**b**) desorption % of AY36 dye ion from NDAC800 using 2.5 g/L of NDAC800 dose and 100 mg/L, *C*_0_ of AY36 dye ion at 25 ± 2 °C.

Regeneration is a commonly utilized method for the adsorption of effluents from water that is critically dependent on the reuse or recycling of adsorbent components, and the economics of this technology are also heavily dependent on this reuse or recycling. The chemical regeneration process was utilized in regenerating the depleted NDAC800. The NDAC800 was regenerated (recycled) by washing it in 0.1 M NaOH solution, 0.1 M HCl solution, clean water at high temperature, and drying it. It was noticed that the maximum adsorption % of AY36 dye ion by NDAC800 was 93.21% after six regeneration rounds (cycles) (Fig. 15a), indicating that activated NDAC800 can regenerate. The maximum desorption % of AY36 dye ion from NDAC800 after six desorption cycles was 92.50% (Fig. 15b).

## Conclusion

We have successfully described an efficient, eco-friendly, cheap and simple method for preparing self-doping activated carbons via hydrothermal and pyrolysis processes at 600, 700 and 800 °C. The NDAC prepared at 800 °C (NDAC800) exhibited a high adsorption capacity of AY36 dye at equilibrium (*Q*_e_ = 211.522 mg/g), and 1.0 g/L of NDAC800 could easily absorb 66.18% of 100 mg/L of anionic dye AY36 within 10 min. In addition, nitrogen atoms were successfully doped into the activated carbon skeleton with nitrogen content 9.85% after carbonization. The specific surface area of NDAC800 reached 727.34 m^2^/g with micropors (mean pore diameter = 1.966 nm). Studies on the NDAC800's surface chemistry showed that the acidic functionality predominated over the basic functionality. NDAC800 possesses a high specific surface area, high nitrogen content and micro-porous materials. The optimum pH value of AY36 dye removal was 1.5, with a removal efficiency of 85.86%. The obtained *Q*_m_ from LIM was 232.522 mg/g. The adsorption kinetics of AY36 dye onto NDAC800 was best described using a PSOM, indicating that the AY36 dye adsorption mechanism onto NDAC800 is governed by chemisorption. The adsorption data were defined well by LIM and TIM. This method can handle the issues from the disposal of dye wastewater and turn the waste, like a fish waste (60% protein) and sawdust, into valuable materials.

## Supplementary Information

Supplementary Figures.

## Data Availability

The datasets used in this investigation are accessible for review upon request from the corresponding author of the paper.
