# Supplementary material for: Fabrication of N-doping activated carbons from fish waste and sawdust for Acid Yellow 36 dye removal from an aquatic environment
Source: Sci Rep. 2023 Apr 11;13:5892. doi: 10.1038/s41598-023-33075-5 (PMC10090169; doi:10.1038/s41598-023-33075-5)
Supplement: Supplementary file 1 — Supplementary Figures. [file 41598_2023_33075_MOESM1_ESM.docx]

**Supplementary**

**Fabrication of N-doping activated carbons from Fish waste and sawdust for Acid Yellow 36 dye removal from an aquatic environment**

Mohamed A. El-Nemr^1^*, Mohamed A. Hassaan^2^, Ibrahim Ashour^1^

^1^Department of Chemical Engineering, Faculty of Engineering, Minia University, Minia 61519, Egypt

^2^Environment Division, National Institute of Oceanography and Fisheries (NIOF), Kayet Bey, El-Anfoushy, Alexandria, Egypt

***** Correspondence: [mohamedelnemr1992@yahoo.com](mailto:mohamedelnemr1992@yahoo.com)

E-mail: [ibrahim.ashour@gmail.com](mailto:ibrahim.ashour@gmail.com) (I.A.); [mhss95@mail.com](mailto:mhss95@mail.com) (M.A.H.)

**
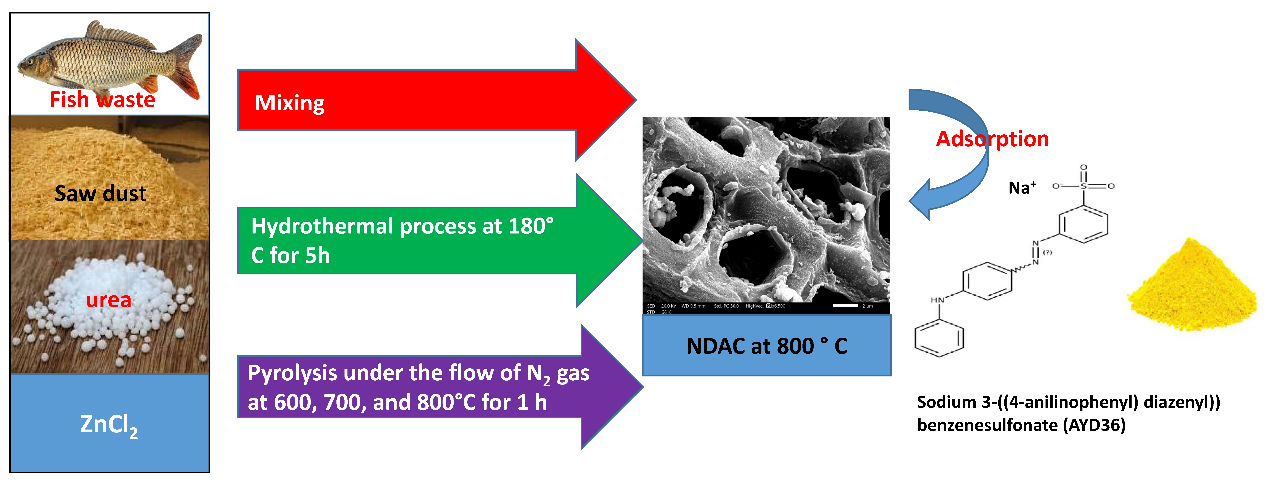
**

Fig. 1S. Preparation steps of NDAC.

|  |  |
| --- | --- |
|  |  |
|  | |

Fig. 2S. Linearized of (a) LIM, (b) FIM, (c) TIM, (d) DRIM, (e) HIM for AY36 dye of in 100-400 mg/L beginning concentration on 1.0 g/L NDAC800 dose at 25±2 °C.

|  |  |
| --- | --- |
|  |  |
|  | |

Fig. 3S. Kinetic of AY36 dye adsorption of 100-400 mg/L beginning concentrations using 1.0 g/L NDAC800 dose at 25±2 °C (a) PFOM; (b) PSOM; (c) EM; (d) IPDM; (e) FDM.
